# Preliminary Experiment Using Sensors for Cow Health Monitoring after Surgical Treatment for the Left Displacement of the Abomasum

**DOI:** 10.3390/s20164416

**Published:** 2020-08-07

**Authors:** Ramūnas Antanaitis, Vida Juozaitienė, Mindaugas Televičius, Dovilė Malašauskienė, Mantvydas Merkis, Eitvydas Merkis, Walter Baumgartner

**Affiliations:** 1 Large Animal Clinic, Veterinary Academy, Lithuanian University of Health Sciences, Tilžės str 18, LT-47181 Kaunas, Lithuania; mindaugas.televicius@lsmuni.lt (M.T.); dovile.malasauskiene@lsmuni.lt (D.M.); eitvydas147@gmail.com (E.M.); 2 Department of Animal Breeding, Veterinary Academy, Lithuanian University of Health Sciences, Tilžės str 18, LT-47181 Kaunas, Lithuania; vida.juozaitiene@lsmuni.lt; 3 Physics Department, Kaunas University of Technology, K. Donelaičio str 73, LT-44249 Kaunas, Lithuania; mantvydas.merkis@ktu.edu; 4 Clinic for Ruminants, University of Veterinary Medicine, Veterinaerplatz 1, A-1210 Vienna, Austria; walter.baumgartner@vetmeduni.ac.at

**Keywords:** experimental sensor, dairy cows, abomasal displacement

## Abstract

The aim of the current study was to determine the effectiveness of two surgical techniques regarding cow respiratory rates, heart rates, and rumination time using two sensors: an experimental device created by the Institute of Biomedical Engineering of Kaunas University of Technology (Lithuania) and the Hi-Tag rumination monitoring system (SCR) produced by SCR Engineers Ltd., Netanya, Israel. The cows were divided into two groups: the PA1 group, containing cows treated by percutaneous abomasopexy (n = 10), and the RSO2 group, containing cows treated by right side omentopexy (n = 8). For the control group (KH), according to the principle of analogs (number of lactations, breed, and days in milk), we selected clinically healthy cows (n = 9). After the surgical treatment for the abomasal displacement, the experimental device was applied for the recording of the heart and breathing rates, 12 h tracking of the rumination time was implemented using the SCR, and the body temperature was measured. After 12 h, the blood was taken for biochemical and morphological tests. With the help of experimental sensors, we found that the more efficient abomasal displacement surgical method was the right side omentopexy: During the first 12 h after right side omentopexy, we found a 5.19 beats/min lower (1.10 times lower) average value of the respiratory rate, a 1.13 times higher level of the heart rate, a 0.15 °C higher temperature, and a 3.29 times lower rumination time compared to the clinically healthy cows. During the first 12 h after percutaneous abomasopexy, we found a 5.19 beats/min higher (1.07 times) average value of heart rate, a 0.02 °C higher temperature, a 6.21 times lower rumination time, and a 0.12 beats/min lower (1.01 times lower) average value of respiratory rate compared to the clinically healthy cows.

## 1. Introduction

Left displacement of the abomasum (LDA) is a condition that occurs primarily in high-producing dairy cows in the postpartum period [1]. LDA causes direct economic loses (correction, medication, discarded milk, etc.) and indirect economic losses (decreased productive and reproductive performances, increased risk of removal from the herd, etc.) [2]. More than 50% of the cases of this disease are diagnosed in the first or the second week of the postpartum period [3]. Feeding high-concentrate diets in early lactation, particularly in high-producing cows, results in a higher susceptibility to LDA. Approximately 90% of cases occur within six weeks following calving. Occasional cases occur in the weeks before birth [4]. LDA is associated with numerous conditions, including stress, metabolic disturbances, and nutritional disorders.

High-milk-production dairy cows that are fed using large quantities of grain with limited exercise may develop abomasal atony [5]. Calcium deficiency has a major influence on the development of abomasal displacement in dairy cows, as aspartate aminotransferase activity was found to increase in all sick dairy cattle. Milk testing data, diet analysis, and regular evaluations of blood serum biochemical indices are the primary criteria for prophylaxis against abomasal displacement in high-producing dairy cattle [6]. In order to return the abomasum to its normal anatomic position, abomasal motility and gas expulsion must be restored. The right paralumbar fossa approach with omental fixation (right paralumbar fossa omentopexy) appears to be the most widely used surgical technique [7]. Omentopexy has been described to be more effective in LDA [7]. Omentopexy and transcutaneous abomasopexy are the most popular methods of surgical treatment; however, there are few studies in the literature regarding which methods are more effective. After surgical treatment of abomasal displacement, it is crucial to monitor the cow’s health status, as the death rate is the highest in the first days after the surgery.

Dairy farmers are in the era of precision farming, which is considered to be important for providing information provision and competing on the market. Sensor systems can effectively and correctly detect illnesses in cows before the milk production is affected. The farm owner can place a sensor onto the cow’s neck, tail, or leg for acquiring real-time data to examine numerous factors such as the cow’s health, behavior, and activity. Sensor-generated data can be used alone or with traditional health-monitoring protocols to identify cows with health disorders [8,9]. The continuous monitoring of behavioral and physiological parameters may allow for the detection of subtle changes before evident clinical signs appear. Earlier disease detection may benefit the cows by preventing progression and improving the response to treatment. An automated rhythm-estimating device would significantly reduce the work required for collecting the required data, as well as increase the frequency of the measurements, allowing the dynamics of rhythm changes to be monitored more closely and the accuracy of the measurements to be increased. This would lead to increased treatment efficacy [10]. In recent years, multiple devices have been developed and implemented by the dairy industry to automatically monitor behavioral and physiological parameters [8,11]. Early identification of cows with health disorders presents opportunities and challenges. Rumination time monitoring can be useful for identifying digestive and metabolic disorders in cows in the early postpartum period [12]. Detecting a health disorder at an early stage, and before the manifestation of clear clinical signs, may benefit the cows by improving the overall treatment response and reducing the negative long-term consequences of disease on their overall health and performance [13,14].

The scientific literature describes a variety of innovative, noninvasive technologies that can be useful for monitoring animal health states and preventing death. The sensor we studied is noninvasive, does not cause pain or stress, operates continuously, and registers the heart and breath rate data over a long period of time. The final rhythm analysis was implemented using a MATLAB environment on a personal computer. The created algorithm used recorded live electrocardiogram (ECG) signals to determine the heart rate. The breathing rate was determined from the chest movement data, which were registered with a piezoelectric sensor. The data for the detection algorithms were acquired from several cows using portable g.MOBIlab+ equipment.

The aim of the current study was to determine the effectiveness of the two mentioned surgical techniques using an experimental device to record the cow respiratory rate, heart rate, and rumination time using two sensors: an experimental device created by the Institute of Biomedical Engineering of Kaunas University of Technology (Lithuania) and the Hi-Tag rumination monitoring system (SCR) produced by SCR Engineers Ltd., Netanya, Israel. 

## 2. Materials and Methods

### 2.1. Location and Experimental Design

The study was performed from September 2018 to December 2019 at the Large Animal Clinic of the Lithuanian University of Health Sciences and at dairy farms in Lithuania. The dairy farm cows were kept free and fed with mixed rations around the year. The feed was well-balanced in order to ensure cow productivity and meet physiological requirements. The main components of the ration were 50% concentrated feed (corn, wheat, and rape), 30% corn silage, 4% hay, and 10% grass silage. The cows were fed two times a day. The breeds of the dairy farm cows were Holstein and its crossbreeds. The average age of the cows was 4.5 years. The mean milk production of the cows was 35 (±5.5) kg/day. LDA was diagnosed according to the auscultation–percussion test, performed during a clinical examination (n = 18). According to the principle of analogs (number of lactations, breed, and days in milk) the cows were divided in to two groups: the PA1 group, containing cows treated by percutaneous abomasopexy (n = 10), and the RSO2 group, containing cows treated by right side omentopexy (n = 8). For the control group (KH), according to the principle of analogs (number of lactations, breed, and days in milk) clinically healthy cows without any clinical symptoms of LDA, ketosis, acidosis, mastitis, metritis, or other diseases after calving were selected (n = 9).

### 2.2. Measurements

After each surgical treatment of the abomasal displacement, the experimental device and SCR sensor were applied. After 12 h, the blood was taken for the biochemical and morphological tests. We made recordings using the SCR 12 h after surgical treatment, for a 12 h length rumination time recording. We also measured the body temperature. The experimental device recorded 12 h of respiratory rate (RR) and heartbeat rate information (HBR). The rumination time was recorded only at the dairy farm because only this farm had a rumination recording system.

After the applied surgical treatment, blood samples for the evaluation of biochemical parameters were taken from the vena (v.) coccygea. The blood specimens were collected using vacuum test tubes (BD Vacutiner, New York, NY, USA. The blood samples were examined in the Large Animal Clinic’s Laboratory of Clinical Tests at the Veterinary Academy of the Lithuanian University of Health Sciences. They were centrifuged with a speed of 3000 rpm for 5 min. The blood serum was examined using blood analyzer Hitachi 705 (Hitachi, Tokyo, Japan) with DiaSys reagents (Diagnostic Systems GmbH, Holzheim, Germany) to determine the concentrations of blood serum β-hydroxybutyrate, calcium, phosphorus magnesium, and iron as well as the activities of aspartate aminotransferase, gamma-glutamyl transferase, iron, phosphorus, and magnesium.

The blood plasma β-hydroxybutyrate concentrations were examined with the Free Style Optium and Medi Sense H systems (Abbott, Maidenhead, UK) using capillary blood from the ear. The blood samples were taken and the β-hydroxybutyrate level was evaluated after the surgical treatment.

### 2.3. Surgical Treatments of LDA

#### 2.3.1. Percutaneous Abomasopexy

Percutaneous abomasopexy was performed for 10 cows. At first, the cow was narcoticized using an intramuscular injection of 1 mL/100 kg body weight xylazine (a xylazine 2% (xylazine hydrochloride) solution for injection, with a withdrawal period of 0 days). The cow’s legs were bound. Then, the cow was laid down on the right flank and was rotated around its axis to the left, pressing down on the suspected place of abomasal displacement. When controlling by auscultation–percussion, the abomasum was restored to its anatomical position, with a tympanic sound at the center of the operating field. The abomasum was fixed using trocars 10 to 15 cm from the xiphoid cartilage and 5 to 7 cm right from the abdomen midline (linea alba). The operative field was prepared, the skin was pulled, and the trocar was attached to the cavity of the abomasum. The abomasum was pierced by a sudden movement. After removing the locking handle and the mandrin, a special surgical suture was placed into the abomasum cavity through the trocar. The trocar was pulled out, and the surgical suture was fixed with a needle stick. Similarly, the second stitch was made. Both ends of the surgical suture were tied in a knot through a special holder. The cow was placed on the sternum.

#### 2.3.2. Right Side Omentopexy

Omentopexy was performed on the right side for eight cows. Initially, a sterile surgical field was prepared (the hair was shaved and the skin was wiped with an antiseptic), and anesthesia was performed: 15 mL of 2% procaine hydrochloride was applied under the processus transversus of the first, second, and fourth lumbar vertebrae, and the incisional infiltration anesthesia was performed. An incision was made 3–5 cm below the lumbar vertebrae. A cut through the skin, subcutaneous tissue, musculus obliquus abdominis internus and externus, musculus transversus abdominis, and peritoneum was performed. Using a sterile needle and a hose, the abomasum was pierced, and the gas was released. The omentum was pulled, and the abomasum was fixed at the duodenum–abomasum junction with nonabsorbable sutures. An additional 5 cm incision was made approximately 15 cm from the knee fold, and the abomasum was attached and fixed by suturing. The surgical wound was sutured on two levels: (1) the peritoneum with the transverse abdominis and obliquus abdominis muscles using a continuous suture and (2) the skin, subcutaneous tissue, and external obliquus muscle.

According our past study, nonsteroidal anti-inflammatory drug (NSAID) injections had a positive effect on the milk yield and cow activity [15]. Caprofen is an analgesic option for dairy cattle following LDA surgery. According to this, all the groups of cows (PA1, RSO2, and KH) were given a subcutaneous injection of Rimadyl Cattle (50 mg/mL) solution for injection (Zoetis, New York, NY, USA at a dose of 1.4 mg per 1 kg body weight (BW) during the surgery.

### 2.4. Sensor for Rumination Time

The Hi-Tag rumination monitoring system (SCR Engineers Ltd., Netanya, Israel) provides output data for the rumination time, intervals between the regurgitation of boluses, and the chewing rate. The system consists of rumination loggers, stationary or mobile readers, and software for processing the electronic records (Data Flow software, SCR Engineers Ltd.). A neck collar was used to position this logger on the left side of the neck. The distinctive sounds produced by regurgitation and rumination were recorded by a microphone, processed, and digitally stored. Every cow had a collar with a rumination sensor on the neck that measured the number of ruminations and sent the data to the computer system to process the data and determine the rumination time. The data were displayed graphically on the computer or mobile application. The rumination time was tracked for a 12 h period.

### 2.5. Experimental Sensor

The experimental sensor accumulated data for the heart rate and breathing rate on a microSD card. The final rhythm analysis was implemented using a MATLAB environment on a personal computer. The created algorithm used the recorded ECG signals to determine the heart rate. The breathing rate was determined from the chest movement data, which was registered with a piezoelectric sensor. The data for the detection algorithms were acquired from several cows using portable g.MOBIlab+ equipment. This equipment was created by the Institute of Biomedical Engineering of Kaunas University of Technology (Lithuania). In the developed algorithm for heart rate detection from the ECG signals, first, the signal was read and filtered with a bandpass filter ranging from 0.67 to 40 Hz in order to remove as much noise as possible from the signal. This ECG frequency range was sufficient for diagnostic purposes, for example, to determine the rhythm of the heart. The received ECG signal was filtered through a moving average filter to remove sudden unwanted peaks in the ECG signal, with minimal effects to the signal itself. After that, signal differentiation was implemented. Since the bandpass filter and moving average filter removed sharp irrelevant peaks in the signal, the R-wave of the ECG signals had the highest derivative value. The R-wave positions were stored and the heartbeat rate was calculated from these. Heart rate values that were over the physiological limits were eliminated as they were possibly associated with movement or other artifacts.

**Figure 1:**
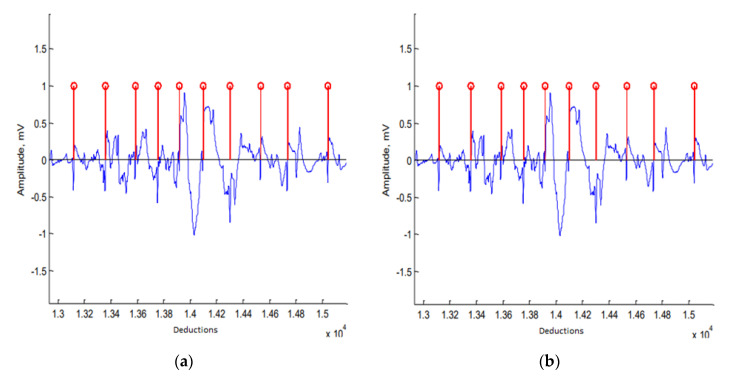
The registered electrocardiogram (ECG) and breathing signals and algorithms according to the maximum systole moments determined using the derivative value (**a**) and algorithm-detected expiration moments (**b**).

The developed algorithm for the heart rate determination performed accurately: The R-wave of ECG signals could be detected even in the case of serous movement artifacts (Figure 1a). The efficiency of the algorithm was confirmed with an additional study where the heart rate was determined using different lead systems. The relative error of the algorithm-determined heart rate (in relation to the manually determined rate) in all cases was less than 1%.

The experimental device also had an installed breathing sensor. The acquired respiration signal was stored on a microSD card. Further rhythm analysis was implemented using a computer. A specialized algorithm was created to determine the respiratory rate from the respiratory curve.

The developed algorithm first acquired the signal from the microSD card, and then the signal was filtered using the bandpass filter at a frequency range of 0.15 to 3 Hz. However, despite filtering, the quality of the signal was very sensitive to animal motion. In order to reduce this dependence, the signal was filtered using a moving average filter. The breathing signal was continuous and changed slowly; thus, filtering with a moving average filter helped to significantly reduce the impact of movement artifacts with minimal damage to the breathing signal. Due to the large number of artifacts in the signal, a standard peak finding function could not be used for the respiratory rate determination. We created a specialized function to search for the maximal value in the positive part of the respiratory signal. The breathing rate was calculated according to the distance between the maximum values (Figure 1b). The respiratory rate values that were over the physiological limits were eliminated as they were possibly associated with the movement of the animal.

The algorithm was tested with the recorded breath signals from several cows at different belt tension levels. When the optimum belt tension level was used, the relative errors of the breath rate determination in relation to the manually determined rate for different subjects were 5.83% and 0.36%. Therefore, we can state that the developed respiration determination algorithm worked appropriately.

### 2.6. Placing the Experimental Special Sensor

An experimental special sensor was placed after the blood collection. First, the animal’s body was prepared for placement of the sensor. At the electrode sites, the skin was cleaned with salicylic alcohol. This was done to remove dirt and grease, which can affect the results.

**Figure 2:**
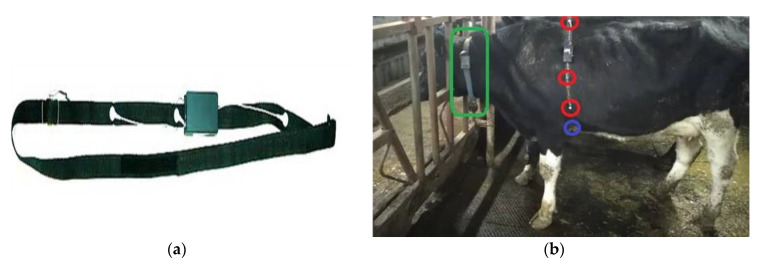
Placing the experimental sensor: sensor electrodes (**a**) were placed on the cow at the gogo, on the left side of the abdominal wall (**b**).

After cleaning, the sensor was placed. The sensor electrodes (Figure 2a) were positioned on the left side of the body. The negative electrode was placed on the left side of the animal behind the caudal portion of the blade, the positive electrode was placed on the left side of the body at the armpit or sternum, and the ground electrode was placed on the left side of the body just below the chest area (Figure 2b). The ground electrode can be placed anywhere on the body, but the gogo area was selected for optimal belt configuration. All of these electrodes were mounted on a belt and formed a line.

An ultrasonic gel (about 5 mL) was applied under the electrodes. The gel was used to reduce air gaps and improve the signal. Parker SignaGel and DERMA-JEL gels were used. Ultrasonic gels were chosen for their ability to dry slowly (within 12 h, as long as the time for the measurements). The belt was tensioned after the gel was applied. The tension must be quite high, because too little tension in the belt results in a large error in the respiratory rate recording. It is also important not to overtighten the belt, because this could break the piezoelectric plate of the recorder.

The final step was to turn on the device. The experimental sensor console had a switch and three indicator lights: registration, unsecured contact, and error. When the device was turned on and the sensor was properly applied, the green light blinked to indicate that the device was operating and registering the respiratory rate and heartbeat rate. A yellow light indicated that there was no proper contact between the electrode(s) and the animal body. The red light indicated that the device had malfunctioned or the battery was discharged. After 12 h, the instrument was switched off. The data were automatically saved to the media.

### 2.7. Data Analysis and Statistics

Samples of the data were analyzed using the SPSS-25 statistical package (version 25.0, IBM, Munich, Germany). The descriptive statistics of data are presented as the mean ± standard error (M ± SEM). The normality of all data recorded in the study was assessed both visually and using the Shapiro–Wilk normality test. We rejected the hypothesis of normality when the *p*-value of the Shapiro–Wilk test was less than or equal to 0.05, and we used nonparametric methods to analyze such indicators (HRB and temperature). The nonparametric Mann–Whitney U test (test for two independent samples) was used for estimation of the significance between values of groups.

A multiple comparison (Bonferroni test) between groups was applied to compare the normally distributed indicators (aspartate aminotransferase, calcium, β-hydroxybutyrate, magnesium, iron, phosphorus, gamma-glutamyl transferase, RR, and rumination time). Spearman’s correlation coefficients were used to determine whether there was a statistically significant relationship between the biochemical and morphological blood indicators and the heart rate, respiratory rate, rumination time, and temperature in different groups of cows. The level of significance was *p* < 0.05 for all tests.

## 3. Results

### 3.1. Biochemical Blood Parameters in Cows

According to the comparative hematological analysis, cows, after the LDA treatment, demonstrated reliably lower concentrations of calcium and phosphorus than clinically healthy cows (Table 1). The lowest levels of calcium phosphorus were found in the RSO2 cows, where they were 1.05–1.24 times lower than in PA1 and 1.41–1.55 times lower than in the KH cows (*p* = 0.001–0.049).

After surgical correction, the PA1 cows showed an increase in aspartate aminotransferase activity (by 2.41–2.42 times) compared with the clinically healthy and RSO2 cows (*p* < 0.001). The highest concentration of β-hydroxybutyrate was observed in cows of the PA1 group, 2.04 times higher compared to KH (*p* = 0.003). The highest gamma-glutamyl transferase activities were found in the PA1 group, 2.32 times higher on average compared to the RSO2 group (*p* = 0.001) and 1.83 times higher compared to the KH cows (*p* = 0.006).

### 3.2. Parameters Measured by Experimental Device by Groups of Cows

The cows of group PA1 demonstrated a 5.19 beats/min higher (1.07 times) average value of HBR, a 0.02 °C higher temperature, a 419.73 min/day lower (6.21 times) rumination time (*p* < 0.001), and a 0.12 beats/min lower (1.01 times) average value of RR compared to the KH cows (Table 2).

The RSO2 cows after surgical correction showed a 5.19 beats/min lower (1.10 times) average value of RR, a 9.44 beats/min higher (1.13 times) level of HBR, a 0.15 °C higher temperature, and 348.20 min/day lower (3.29 times) rumination time (*p* < 0.001) compared to the group of KH cows.

### 3.3. Relationship between the Traditional Blood Biochemical and Morphological Parameters and the Parameters Measured by the Experimental Device

The data of the correlation analysis by cow group are given in Table **3**. The cow temperature was statistically significantly negatively associated with β-hydroxybutyrate (*r* = −0.812, *p* < 0.01) in the KH group and with gamma-glutamyl transferase (*r* = −0.753, *p* < 0.05) in the RSO2 group. In the PA1 group, we found the highest temperature correlation coefficients for calcium (*r* = 0.624) and phosphorous (*r* = 0.596); however, these coefficients were not significant (Table 3).

## 4. Discussion

Abomasal displacement is the one of the most important organic and metabolic disorders of cows [16]. Veterinarians are not sufficiently provided with equipment to monitor animal vital signs. The most important vital signs are the respiratory rate and heart rate. Sensors can be useful for remote evaluation of cows, in particular when a farmer or veterinarian must look after many of them. The diseased cows were afebrile with increased heart and respiratory rates and reduced ruminal movements. These findings conformed to those of Goetze and Müller [17] and El-Attar et al. [18]. The signs of hypovolemic shock (increased heart rate and dehydration) and endotoxemia, along with evidence of a shift from hypokalemic hypochloremic metabolic alkalosis to a high-anion-gap metabolic acidosis (lactic acidosis), are common indicators of abomasal displacement. A heart rate higher than the normal range could be due to dehydration and electrolyte changes, including hypokalemia [17]. In the current study, after the surgical treatment for the abomasal displacement, the experimental device was applied for the recording of the heart and breathing rates, 12 h tracking of the rumination time was implemented using the SCR, and the body temperature was measured. After 12 h, the blood was taken for biochemical and morphological tests.

RSO2 cows after surgical correction showed a 5.19 beats/min lower (1.10 times) average value of RR, a 9.44 beats/min higher (1.13 times) level of HBR, a 0.15 °C higher temperature, and a 348.20 min/day lower (3.29 times) rumination time (*p* < 0.01) compared to the group of KH cows. Dezfouli et al. [16] reported that the ill cows were feverless but had increased respiratory and heart rates, and a reduction of ruminal movements was noted. This was also confirmed by the investigations of El-Attar et al. [18]. An abnormal temperature, pulse, and respiratory rate were typically a response to other conditions, such as metritis or mastitis. Stangaferro et al. [12] reported dramatic reductions in rumination before the occurrence of ketosis and LDA in dairy cows. As Stangaferro et al. [12] suggested, physical activity and rumination time monitoring can be useful for identifying digestive and metabolic disorders for cows in the early postpartum period. Automated health-monitoring systems, which use rumination and activity, are promising tools for the identification of cows with metabolic and digestive disorders in dairy farms [12].

Cows with LDA have increased respiratory and heart rates as well as reduced ruminal movements. Reduced ruminal movements are associated with hypocalcemia, which is common with LDA. Newby et al. [19] reported increased respiratory and heart rates in cattle with displaced abomasum. They stated that respiratory and heart rates could be potential postoperative pain indicators and that increased heart and respiratory rates were associated with abdominal pain. The respiratory rate also increases in patients with pneumonia, and pain in the chest, in the anterior part of the abdominal cavity. An increase of the respiratory rate is also one of the best indicators of stress. Tachycardia occurs when animals are frightened, and also with infectious, metabolic, and toxic diseases. Tachycardia is also one of the main indicators of pain. Bradycardia is rare in cattle. It is associated with pituitary abscesses, botulism, and vagus nerve lesions. Boulay et al. [20] stated that the heart rate is an objective prognostic indicator after abomasum displacement correction surgery. An increased heart rate is associated with a decreased chance of recovery after surgery. Research determined that 56% of cattle with abomasum displacement, volvulus, and more than 100 beats/min heart rate were euthanized, salvaged, or died in the course of treatment compared to only 12–25% when the heart rate was lower than 100 beats/min.

The results of the study demonstrated a significant reduction in the Ca levels for cows after the LDA treatment. According to the literature, severe hypocalcemia was associated with decreased abomasal motility [21].

The highest concentration of β-hydroxybutyrate was observed in cows of the PA1 group—2.04 times higher compared to KH (*p* = 0.003) and 1.35 higher compared to cows of the RSO2 group. Cows with displaced abomasum were observed to develop hepatic lesions and ketosis after the surgical treatment was applied [22]. LDA cows have an increased risk of severe ketosis and metritis [4]. According LeBlanc et al. [23], the negative energy balance during the postpartum period is one of the key aspects in the pathogenesis of LDA. The circulating concentrations of non-esterified fatty acids and β-hydroxybutyrate during the peripartum period are related to the timing and magnitude of this period. These parameters are associated with an increased risk of abomasal displacement.

After surgical correction, the PA1 cows showed an increase in the aspartate aminotransferase concentration (2.41–2.42 times) compared with clinically healthy and RSO2 cows (*p* < 0.001). A significant increase in aspartate aminotransferase and alkaline phosphatase was noted in cows with LDA. Similar results were acquired by Zadnik [24]. In an investigation of Antanaitis et al. [22], a 158 IU/L average aspartate aminotransferase activity in cows with LDA was determined, while in the control group this was 86.9 IU/L.

The highest gamma-glutamyl transferase values were found in the PA1 group, at 1.83–2.32 times higher on average compared to the RSO2 group (*p* < 0.01) and 1.83 times higher when compared to the KH cows (*p* < 0.01).

The serum gamma-glutamyl transferase activity in cattle with abomasum displacement allowed us to determine the presence of cholestatic disorders with a relatively higher sensitivity compared to the serum alkaline phosphatase activity. The increased serum gamma-glutamyl transferase activity in calves indicated that species that produce large amounts of gamma-glutamyl transferase activity in the mammary glands can excrete gamma-glutamyl transferase in the colostrum. The increased mean enzyme activities of aspartate aminotransferase and gamma-glutamyl transferase indicated associations of abomasum dislocation with hepatocyte damage, endotoxemia, and hepatic lipidosis. Zadnik [24] reported that distributed outflow to the duodenum could indicate significantly increased gamma-glutamyl transferase activity.

## 5. Conclusions

For cows that required postsurgical aftercare, we used a sensor to measure the heart rate, respiratory rate, and rumination time to determine the risks of not providing sufficient postoperative care. With the help of experimental sensors, we found that right side omentopexy was the more efficient abomasal displacement surgical method: during the first 12 h after right side omentopexy, we found a 5.19 beats/min lower (1.10 times) average value of the respiratory rate, a 1.13 times higher level of the heart rate, a 0.15 °C higher temperature, and a 3.29 times lower rumination time compared to the clinically healthy cows. During the first 12 h after percutaneous abomasopexy, we found a 5.19 beats/min higher (1.07 times) average value of heart rate, a 0.02 °C higher temperature, a 6.21 times lower rumination time, and a 0.12 beats/min lower (1.01 times) average value of respiratory rate compared to the clinically healthy cows.

In future research, the accuracy of experimental sensors for practical applications can be improved by using the abomasal displacement surgical methods with a longer experimental period (more than 12 h after surgery).

## Figures and Tables

**Table 1:** The biochemical blood parameters and mineral content for control (KH), percutaneous abomasopexy (PA), and right side omentopexy (RSO) groups of cows.

Blood Indicators	KH	PA1	RSO2	*p*-Values		
				KH-PA1	KH-RSO2	PA1-RSO2
Aspartate aminotransferase (UV/L)	53.33 ± 9.825	128.3 ± 9.321	53.00 ± 10.430	<0.001	0.999	<0.001
Calcium (mmol/L)	2.28 ± 0.135	1.82 ± 0.128	1.47 ± 0.143	0.049	0.001	0.245
β-hydroxybutyrate mmol/L	0.47 ± 0.095	0.96 ± 0.090	0.71 ± 0.100	0.003	0.262	0.235
Magnesium (mmol/L)	0.82 ± 0.223	0.88 ± 0.212	1.14 ± 0.237	1.000	1.000	1.000
Iron (µmol/L)	22.04 ± 4.645	15.28 ± 4.407	24.24 ± 4.927	0.905	1.000	0.564
Phosphorus (mmol/L)	2.07 ± 0.101	1.55 ± 0.096	1.47 ± 0.107	0.003	0.001	1.000
Gamma-glutamyl transferase (UV/L)	13.54 ± 2.332	24.79 ± 2.213	10.67 ± 2.474	0.006	1.000	0.001

**Table 2:** The heartbeat rate (HBR), respiratory rate (RR), rumination time, and temperature for each group of cows.

Indicator	KH	PA1	RSO2	*p*-Values		
				KH-PA1	KH-RSO2	PA1-RSO2
RR (beats/min)	32.56 ± 1.418	32.40 ± 1.346	29.63 ± 1.504	1.000	0.508	0.546
HBR (beats/min)	71.56 ± 3.832	76.75 ± 4.251	81.00 ± 4.440	0.269	0.123	0.450
Rumination time (min/day)	500.33 ± 22.070	80.60 ± 20.937	152.13 ± 23.409	<0.001	<0.001	0.096
Temperature (°C)	38.80 ± 0.111	38.82 ± 0.090	38.95 ± 0.142	0.805	0.383	0.394

**Table 3:** The heart rate, respiratory rate, rumination time, and temperature correlation coefficients with blood indicators for each group of cows.

Blood Indicators	KH	PA1	RSO2	KH	PA1	RSO2
	RR			HBR		
Aspartate aminotransferase	0.629	−0.090	−0.024	−0.473	0.177	−0.476
Calcium	0.035	−0.172	0.072	0.119	0.389	0.214
β-hydroxybutyrate	−0.009	−0.430	−0.825	0.053	−0.074	−0.335
Magnesium	0.547	−0.345	0.072	0.067	−0.012	0.381
Iron	0.323	−0.086	−0.491	−0.050	−0.231	−0.024
Phosphorus	−0.284	−0.384	0.359	0.145	0.410	0.310
Gamma-glutamyl transferase (GGT)	0.562	−0.055	−0.247	−0.217	0.243	−0.168
	Rumination time			Temperature		
Aspartate aminotransferase	0.093	−0.389	−0.048	−0.644	−0.025	−0.347
Calcium	0.254	0.127	−0.238	0.086	0.624	0.491
β-hydroxybutyrate	0.186	0.497	−0.132	−0.812 **	0.225	−0.181
Magnesium	0.544	−0.006	−0.190	0.128	−0.148	0.252
Iron	−0.583	0.006	0.119	−0.339	0.531	0.108
Phosphorus	0.299	0.055	−0.452	−0.200	0.596	0.395
Gamma-glutamyl transferase	0.583	0.248	0.299	−0.034	0.519	−0.753 *

* Spearman’s correlation coefficient between indicators is significant at the level *p* < 0.05. ** Spearman’s correlation coefficient between indicators is significant at the level *p* < 0.01.
