# Correction: Antanaitis, R., et al. Preliminary Experiment Using Sensors for Cow Health Monitoring after Surgical Treatment for the Left Displacement of the Abomasum. *Sensors* **2020**, *20*, 4416

**DOI:** 10.3390/s21030918

**Published:** 2021-01-29

**Authors:** Ramūnas Antanaitis, Vida Juozaitienė, Mindaugas Televičius, Dovilė Malašauskienė, Mantvydas Merkis, Eitvydas Merkis, Walter Baumgartner

**Affiliations:** 1 Large Animal Clinic, Veterinary Academy, Lithuanian University of Health Sciences, Tilžės str 18, LT-47181 Kaunas, Lithuania; mindaugas.televicius@lsmuni.lt (M.T.); dovile.malasauskiene@lsmuni.lt (D.M.); eitvydas147@gmail.com (E.M.); 2 Department of Animal Breeding, Veterinary Academy, Lithuanian University of Health Sciences, Tilžės str 18, LT-47181 Kaunas, Lithuania; vida.juozaitiene@lsmuni.lt; 3 Physics Department, Kaunas University of Technology, K. Donelaičio str 73, LT-44249 Kaunas, Lithuania; mantvydas.merkis@ktu.edu; 4 University Clinic for Ruminants, University of Veterinary Medicine, Veterinaerplatz 1, A-1210 Vienna, Austria; walter.baumgartner@vetmeduni.ac.at

The authors wish to make the following corrections to this paper [1]: in the original version of our article, insufficient acknowledgement was given for the source of some of the statement in the materials and methods section, Section 2.4.

We apologize for the original error. To correct this oversight, Schirmann et al., 2009 [2] has now been inserted in Section 2.4, Paragraph 1 and should read: The Hi-Tag rumination identification system (SCR Engineers Ltd., Netanya, Israel) registers data of rumination time, chewing rate and time intervals between regurgitation of boluses. The system operates with the use of rumination loggers, mobile or stationary readers, and software for processing of the electronic records (Data Flow software, SCR Engineers Ltd.). The logger is mounted on a neck collar, that positions it on the left side of the neck [16].

The authors apologize for any inconvenience caused and state that the scientific conclusions are unaffected. The original article has been updated.
